# Phase-Dependent Electrochemical Performance of Co_x_S_y_ (x = 1,9; y = 2,8) for Symmetric Supercapacitor Application

**DOI:** 10.3390/ma18092101

**Published:** 2025-05-03

**Authors:** Ankush Sharma, Young-Bin Cho, Tung Bach Tran, Sung Jin Kim, Dong In Park, Taehoon Kim, Vishwa Bhatt, Manjeet Kumar, Ju-Hyung Yun

**Affiliations:** 1 Department of Electrical Engineering, Incheon National University (INU), 119 Academy-ro, Yeonsu-gu, Incheon 22012, Republic of Korea; ankush@inu.ac.kr (A.S.); bachtt@inu.ac.kr (T.B.T.); 2 Water Environmental Analysis Center, Korea Testing & Research Institute (KTR), Gwacheon 13810, Republic of Korea; 3 Department of Electrical and Computer Engineering, University of Louisville, Louisville, KY 40292, USA; sungjin.kim@louisville.edu; 4 Micro/Nano Technology Center, University of Louisville, Louisville, KY 40292, USA; 5 Department of Safety Engineering, Incheon National University (INU), 119 Academy-ro, Yeonsu-gu, Incheon 22012, Republic of Korea; sunydong@inu.ac.kr (D.I.P.); kths@inu.ac.kr (T.K.); 6 Department of Chemical and Biochemical Engineering, Dongguk University, Pildong-ro 1-gil, Jung-gu, Seoul 04620, Republic of Korea; vbhatt23@dgu.ac.kr

**Keywords:** electrochemical energy storage (EES), transition metal sulfides, hydrothermal, symmetric supercapacitor, CoS_2_, Co_9_S_8_

## Abstract

Modulating the oxidation states of transition metal species is a practical approach to enhance redox activity and increase the number of active sites in electrode materials. Herein, we describe a simple one-step hydrothermal approach to prepare Co_x_S_y_ with two different phases, cobalt pyrite (CoS_2_) and cobalt pentlandite (Co_9_S_8_), to explain the influence of material microstructure and properties on electrochemical performance. The as-prepared CoS_2_ and Co_9_S_8_ were investigated as symmetric supercapacitor (SC) devices for potential energy storage applications. Co_9_S_8_ exhibited the highest specific gravimetric capacitance of 14.12 Fg^−1^ at 0.2 mAcm^−2^ with capacitance retention of 91.3% after 10,000 cycles, indicating robust cycling stability. In addition, the Co_9_S_8_ SC device showed the highest energy (E) and power (P) density of 9.14 Whkg^−1^ and 0.23 kWkg^−1^. These results highlight a simple approach of tailoring different phase syntheses of Co_x_S_y_ structure toward high-performance electrode material for energy storage and conversion.

## 1. Introduction

Electrical energy demand in the modern world has grown exponentially due to the increased human population and its dependency on electronic devices [1,2]. Climate change restricts the utilization of renewable energy sources in the continuous endeavor to reduce reliance on fossil fuels. The unpredictable behavior of solar, wind, and tidal energy production has prompted the quest for dependable energy storage solutions to stabilize electricity supply and demand [3,4]. In the discipline of materials and electrical engineering, electrochemical energy storage (EES) technologies are at the forefront, demonstrating significant potential in the global initiative to tackle the challenges associated with sustaining the energy supply. EES devices, which include SCs and batteries, can effectively store charges, facilitating the extraction and transformation of renewable energy into a usable form. Nonetheless, these devices possess both strengths and weaknesses [5,6,7,8,9]. A SC represents an EES device, integrating the benefits of both batteries and conventional capacitors [10,11].

The active electrode material is widely recognized as a crucial factor influencing the electrochemical performance of SCs. In recent years, transition metal sulfides (TMSs) have emerged as significant contributors to the advancement of energy storage and conversion technologies in SCs [12,13,14,15,16,17]. The 3d orbitals of transition metal atoms remain partially filled, resulting in the presence of multiple valence states. In redox reactions that utilize pseudocapacitance for charge storage, an increase in the valence states of metal atoms leads to a higher generation of electrons during the reaction process. This, in turn, results in enhanced stored energy and elevated specific capacitance. In comparison to conductive polymers, transition metal oxides, and hydroxides, TMSs exhibit superior electrochemical activity [18,19]. Nonetheless, TMSs tend to agglomerate easily, and they are susceptible to irreversible damage from lattice distortion resulting from phase transformation, which impacts the stability of the electrode materials [20].

When compared to their cobalt hydroxide or oxide counterparts, cobalt sulfides exhibit more enhanced electrical conductivity, extensive redox reactions, and greater theoretical specific capacitance (5449 F/g) [21,22]. The extensive redox chemistry provides them with a greater capacity compared to many metal oxides and carbon materials. Zhou et al. developed a multilayer-stacked Co_9_S_8_ microstructure on carbon cloth using a straightforward chemical solution processing method, achieving an impressive specific capacitance of 1475.4 Fg^−1^ [23]. Rathinamala et al. fabricated a CoS_2_/MWCNT architecture and achieved a specific capacitance of 524 Fg^−1^ [24]. Nevertheless, the majority of the reported works exhibit low specific capacitances and inadequate cycling performance, necessitating further research on the development of TMS-based SC electrode materials. Consequently, to fulfill the demands of practical applications, additional efforts are necessary to develop easy, efficient, and sustainable methods for the fabrication of cobalt sulfides electrode materials with improved capacitance and cyclic stability.

Based on the above discussion, multiphase cobalt sulfides (CoS_2_ and Co_9_S_8_) were synthesized using a simple one-step hydrothermal approach. The multiphase cobalt sulfides were coated on Ni-Foam (Ni-F) and utilized as SC electrodes. The Co_9_S_8_ electrode showed the highest capacitance of 14.12 Fg^−1^, surpassing cycling durability with capacitance retention of 91.3% over 10,000 cycles. This can be attributed to the capability of Co_9_S_8_ to produce abundant redox reactions and excellent electrical conductivity. Therefore, the present study provides an in-depth analysis of multiphase cobalt sulfide for SC application.

## 2. Materials and Methods

### 2.1. Reagents

All reagents were commercially available and used without further purification. Cobalt acetate tetrahydrate [(CH_3_COO)_2_Co.4H_2_O, 98%], thioacetamide (CH_3_CSNH_2,_ 98%), thiourea (NH_2_CSNH_2_, 98%) were acquired from Junsei Chemical and Sigma Aldrich, respectively. Hydrochloric acid (HCl), isopropyl alcohol (IPA) [(CH_3_)_2_CHOH], and ethyl alcohol (EtOH) were provided by Sigma and Daejung chemicals.

### 2.2. Synthesis of Co_x_S_y_

#### 2.2.1. Synthesis of CoS_2_

**Figure 1:**
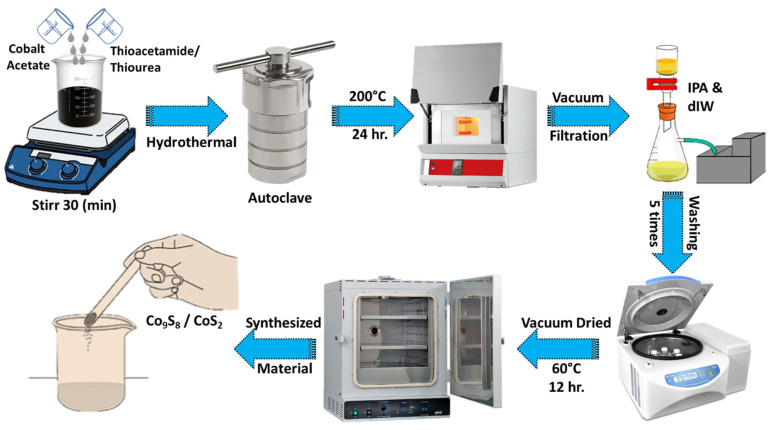
Schematic illustration depicting synthesis process of Co_x_S_y_ nanostructure.

Initially, (CH_3_COO)_2_Co.4H_2_O (0.1M), and NH_2_CSNH_2_ (0.2 M) were continuously stirred into 80 mL, 5:5 (*v*/*v*) of EtOH and deionized water (dIW), for half hour at room temperature as shown in Figure 1. The resulting suspension was hydrothermally heated at 200 °C for 24 h and allowed to cool down to room temperature under ambient conditions. The solid obtained *CoS_2_* was washed with IPA and dIW and dried at 60 °C for 12 h.

##### 2.2.2. Synthesis of Co_9_S_8_

A mixture of (CH_3_COO)_2_Co.4H_2_O (1.125 mM), CH_3_CSNH_2_ (11.25 mM), and dIW (80 mL) was continuously stirred for half an hour at room temperature. The mixed solution was sealed in a 100 mL Teflon-lined stainless-steel autoclave and heated at 200 °C for 24 h. Subsequently, the autoclave was cooled to room temperature. The solid Co_9_S_8_ crystals were collected and vacuum filtered with IPA and dIW and dried at 60 °C for 12 h.

### 2.3. Material Characterization

The X-ray powder diffraction (XRPD) data were recorded on a SmartLab High-resolution X-raydiffractometer (Rigaku, Japan) using Cu Kα with an acceleration voltage of 45 kV and 2θ ranging from 20° to 80°. The morphological investigation was performed using a scanning field emission electron microscope (FE-SEM, JEOL JSM-7800F, 15 kV) and transmission electron microscope (FE-TEM, Thermo Fisher Scientific Talos F200X, 200 kV). X-ray photoelectron spectroscopy (XPS) was carried out using ULVAC-PHI 5000 Versa Probe II to examine the chemical composition of prepared materials.

### 2.4. Assembly of Symmetric Supercapacitor

**Figure 2:**
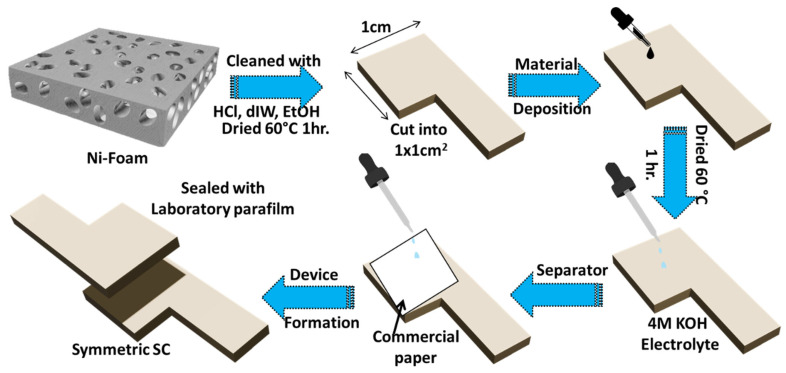
Schematic illustration of the fabrication of a symmetric SC device.

The working electrode was prepared by using a simple drop-casting technique. The required slurry was prepared using active material, activated carbon, and PVDF in a weight ratio of 7:1:2 in NMP. The obtained slurry (0.01 gcm^−2^) was coated onto Ni-Foam (NiF) substrate with an active area of 1 × 1 cm^2^ and dried at 60 °C for 12 h. Before that, the NiF substrate was cleaned by ultrasonication in 1 M HCl solution for 15 min., washed with dIW and EtOH, and vacuum dried at 60 °C for 1 h. After the slurry was coated, electrodes were put on each other, separated by using a commercial filter paper as a separator, and covered with 4 M KOH electrolyte. Afterward, the device was sealed with laboratory parafilm, resulting in a symmetric supercapacitor (SC) assembly as shown in Figure 2.

### 2.5. Electrochemical Measurement

The electrochemical characteristics were measured at room temperature using Ivium Technologies V74635 potentiostat–galvanostat with a robust selection of electrochemical analytical methods via exploration of the cyclic voltammetry (CV), a galvanostatic charge–discharge test (GCD), and the electrochemical impedance spectroscopy (EIS) technique. The CV measurements were carried out at different scan rates between 5 and 1000 mV/s within a voltage window of 0 V to 0.6 V. The GCD tests were also performed under a range of current density from 0.2 to 0.6 mAcm^−2^, within the same voltage window. Furthermore, EIS measurements were conducted in the frequency range of 0.01 hZ to 100 kHz to measure the kinetics and mechanistic of the electrochemical system. Equations (A1)–(A3) provide the specific capacitance of the cell (C_cell_), energy (E_d_), and power density (P_d_) for the two-electrode system based on the GCD [25,26,27].

## 3. Results

### 3.1. Physiochemical Examination

**Figure 3:**
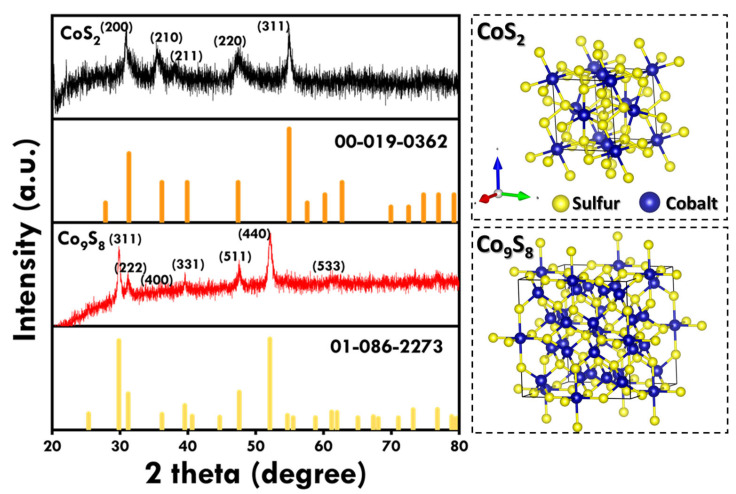
XRD patterns of as-synthesized CoS_2_ and Co_9_S_8_ nanostructures and their corresponding crystal structure.

The phase purity, crystalline structure, and composition of the hydrothermally fabricated samples were confirmed using XRD patterns, as shown in Figure 3. The observed peaks align with the two different phases of Co_x_S_y,_ namely CoS_2_, and Co_9_S_8_. The XRD pattern of CoS_2_ exhibited strong and sharp peaks at 2θ = 30.9° and 54.98°, corresponding to reflections from crystal planes (200) and (311). Other peaks of low intensity were observed at around 2θ = 27.89°, 35.56°, 38.22°, 47.38°, and 63.79°, corresponding to reflections from the (111), (210), (211), (220), and (321) planes. The observed peaks align with the JCPDS No. 00-019-0362 [28], having a cubic crystal system and the Pa3 space group. For Co_9_S_8,_ the sharp and strong peaks were observed at 2θ = 29.82° and 52.13°, corresponding to reflections from the (311) and (440) planes. Other peaks were observed at 2θ = 15.4°, 31.2°, 36.18°, 39.64°, 47.56°, 52.13°, and 61.2° corresponding to reflections from the (111), (222), (400), (331), (511), (440), and (533) planes. The observed peaks align with the JCPDS No. 01-086-2273 [29], having a cubic crystal system and Fm-3m space group. The absence of additional diffraction peaks verifies the phase purity of the synthesized material.

**Figure 4:**
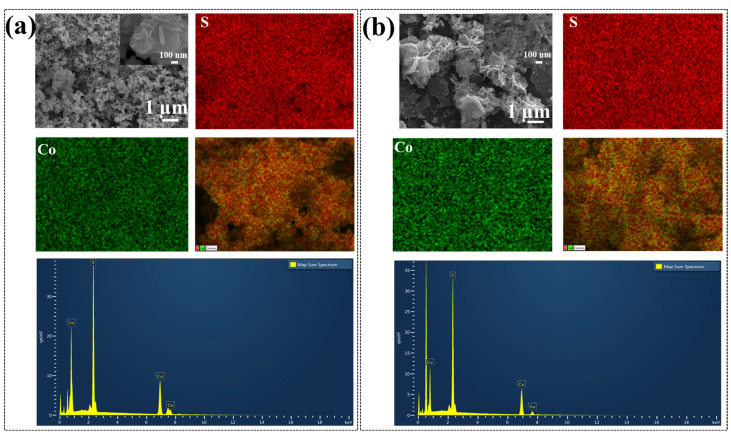
FE-SEM images, EDS spectra, and elemental mapping of (**a**) Co_9_S_8_ and (**b**) CoS_2_ nanostructure.

Furthermore, FE-SEM was used to investigate the structural and morphological evaluation with the change in the phase of Co_x_S_y_. The morphologies of the as-prepared samples are shown in Figure 4a,b under various magnifications. From the Figure 4a, it is observed that the Co_9_S_8_ hierarchical structure exhibits flakes-type morphology and CoS_2_ exhibits hierarchical flower-type morphology (Figure 4b). Flower-type morphology can be attributed to the self-assembled nanosheets arrangement in different orientations. The growth mechanism of nanoflowers can be attributed to the Ostwald ripening process [30]. The difference in morphology can be attributed to the different solvents used in the synthesis process, confirming the significant effect of the binary solvent mixture on the formation of different phases of cobalt sulfides. Dong et al. also studied systematically the solvent interaction in detail and confirmed the hierarchical structure evolution in cobalt sulfide nanostructures [31]. From the ImageJ version-1.54g analysis, it was found that the Co_9_S_8_ flakes size ranges from 200 to 800 nm, while the CoS_2_ flower-like petals length ranges from 1.4 to 3.6 µm. Figure 4 shows the SEM-energy dispersive X-ray spectroscopy (EDS) mapping results of both structures. It can be seen that Co and S are distributed uniformly. The presence of Co and S from the elemental mapping spectrum can be observed, suggesting that the two elements are present in the structure, as shown in Figure 4.

**Figure 5:**
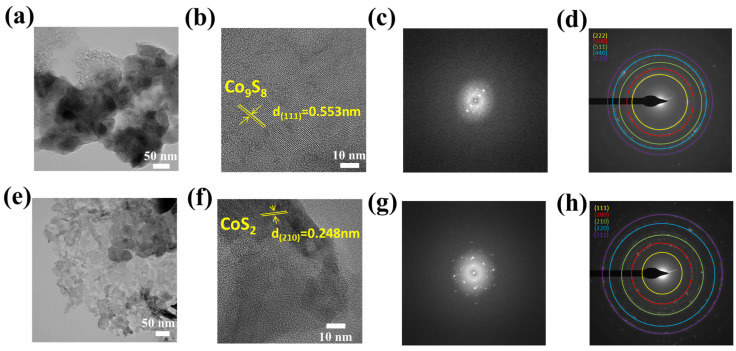
TEM images, HR-TEM images, FFT, and SAED pattern of (**a**–**d**) Co_9_S_8_ and (**e**–**h**) CoS_2_ nanostructure.

Furthermore, TEM micrographs have been recorded, as shown in Figure 5a,e. TEM images confirm the layered structure of flakes for the Co_9_S_8_ and CoS_2_ nanoflower hierarchical structure. Figure 5b,f show the higher resolution and magnification image of the flakes and flowers, depicting the stack-layered orientation of the nanosheets. The lattice fringes with d spacing of 0.553 nm ascribed to the (111) crystal plane of cubic Co_9_S_8_ and d-spacing of 0.248 nm ascribed to the (210) crystal plane of cubic CoS_2_. These arrangements were in agreement with the XRD analysis. Additionally, the HRTEM images shown in Figure 5b,f have been filtered using the fast Fourier transform (FFT), as shown in Figure 5c,g. The FFT pattern of the Co_9_S_8_ and CoS_2_ samples displays the paired bright spots ascribed to the crystalline nature of each sample. Figure 5d,h show the FFT-generated scanning area electron diffraction (SAED) pattern for Co_9_S_8_ and CoS_2_, well indexed to cubic structure and polycrystallinity. The above results are in good agreement with the above-stated XRD results.

**Figure 6:**
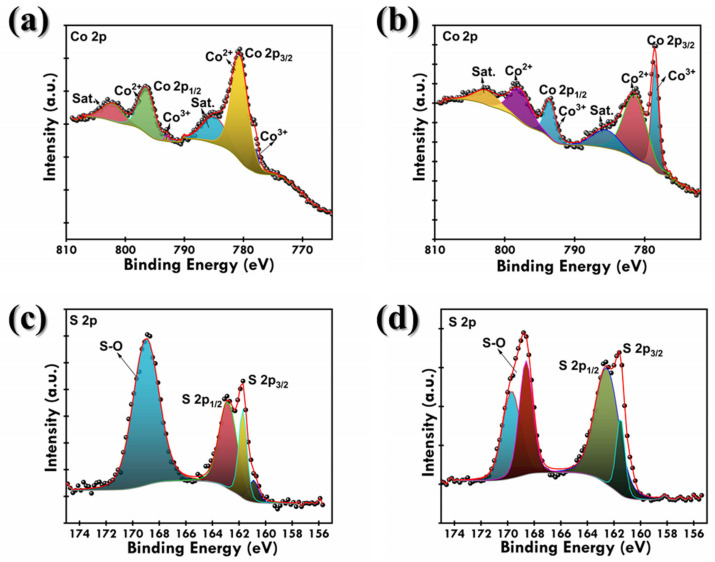
High-resolution S 2p and Co 2p XPS spectra of (**a**,**c**) Co_9_S_8_ and (**b**,**d**) CoS_2_.

**Figure A1:**
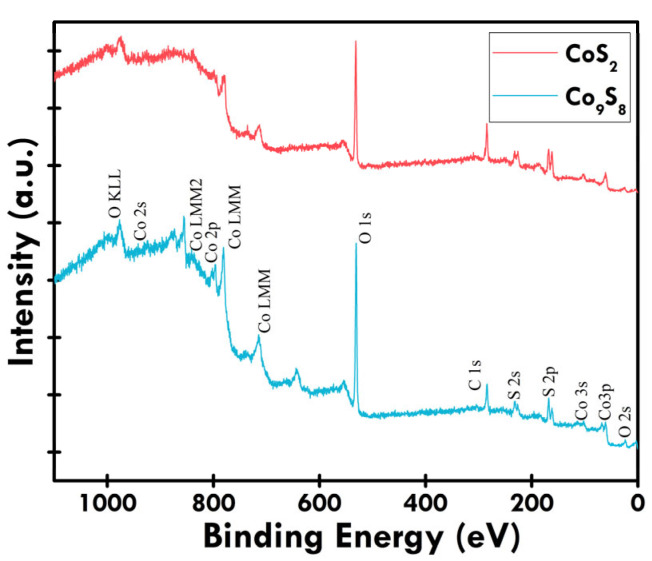
XPS survey spectra for CoS_2_ and Co_9_S_8_.

To attain the in-depth understanding of chemical states for the as-synthesized samples, XPS studies were performed. The XPS observations were carried out in a 2.4 × 10^−9^ Torr vacuum. To remove the inelastic background, the Shirley function was used. The whole data were referenced to the C 1s peak, 284.9 for the samples. Figure A1 displays the XPS survey spectra, and Figure 6 displays the core-level spectra for both the samples. In the XPS survey spectra, Co, S, and O are observed in the samples. The presence of the O element can be attributed to the slight surface oxidation and adsorbed O_2_ and water. The high-resolution S 2p XPS peaks revealed the presence of S-O (168.9 eV), S 2p_1/2_ (162.8 eV), and S 2p_3/2_ (161.7 eV) (Figure 6c,d) [32,33]. The S-O can be ascribed to the partial oxidation of S; in addition, the Co-S peak originated from Co_9_S_8_. Also, the S 2p peaks are more intense in CoS_2_, indicating its S-rich nature [34]. The S-O contribution is slightly more pronounced in CoS_2_, which may deteriorate its chemical performance by forming insulating layers.

As shown in Figure 6a,b, both the samples show similar features, of which Co 2p_3/2_ spectra arise from spin-orbit characteristics of Co^3+^ and Co^2+^ at lower binding energy and Co 2p_1/2_ spectra originating from spin-orbit characteristic of Co^3+^ and Co^2+^ at higher binding energy, confirming the coexistence of Co^3+^ and Co^2+^ cations in cobalt sulfides [35,36]. Along with these two shakeup satellites, peaks are also observed (identified as Sat.). Due to the complex crystal structure of Co_9_S_8_, the coexistence of both oxidation states is observed and is generally broader as compared to sharper Co 2p peaks in CoS_2_. Along with this, the peaks in CoS_2_ appear more distinct and better separated due to the dominant Co^2+^ oxidation state. These differences highlight the mixed valence in Co_9_S_8_ and the more defined Co^2+^ environment in CoS_2_, which can be attributed to their different chemical compositions. The relatively higher intensity ratio of Co^3+^ to Co^2+^ in Co_9_S_8_ compared to CoS_2_ suggests that Co_9_S_8_ possesses a larger fraction of Co^3+^ species. Co^3+^ ions are known to be electrochemically more active because they can easily participate in redox reactions, thus enhancing pseudocapacitive behavior. On the other hand, CoS_2_ shows a dominant presence of Co^2+^ species. The dominance of Co^2+^ limits the extent of reversible faradaic reactions, resulting in inferior capacitance compared to Co_9_S_8_.

### 3.2. Electrochemical Examination

**Figure 7:**
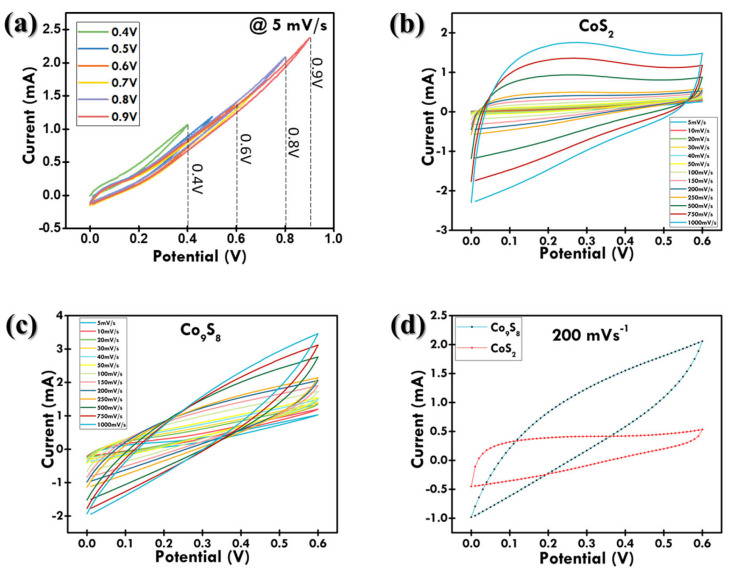
(**a**) CV showing OPW test between 0.4–0.9 V; CV measurements at scan range from 5 to 1000 mV/s for (**b**) CoS_2_ and (**c**) Co_9_S_8_; (**d**) comparative CV curves at 200 mV/s of CoS_2_ and Co_9_S_8_ symmetric SC cell.

To investigate the charge-storage properties of the as-prepared symmetric SC device, a two-electrode cell setup using 4 M KOH aqueous ionic liquid as the electrolyte was employed. An operating potential window (OPW) test was performed to determine the voltage range within which the device can safely operate without causing failure, degradation, and irreversible chemical reactions. CV was used by sweeping the potential from low to high voltage (0.4–0.9 V), as shown in Figure 7a. After 0.6 V, a sharp increase in current was observed, indicating electrolyte decomposition. So, 0.6 V was decided as the maximum OPW for the cell.

CV measurements were performed at different scan rates, ranging from 5 to 1000 mV/s with the potential range fixed between 0 and 0.6 V, as depicted in Figure 7b,c. From the CV curves, it was observed that the cell displayed pseudocapacitive behavior, as both CoS_2_ and Co_9_S_8_ are transition metal sulfides, which are known for their ability to exhibit pseudocapacitive behavior due to reversible redox reactions. This observed pseudocapacitive behavior is advantageous for applications requiring rapid energy delivery and high-power density, such as portable electronics, hybrid electric vehicles, and power backup systems. The ability to store and release charge quickly without significant loss in performance makes pseudocapacitive materials highly suitable for these applications. The redox reactions that occurred on the electrode surface can be expressed as Equations (A4)–(A7).

According to Figure 7b,c, the increase in scan rates results in the shifting of oxidation and reduction peaks, towards higher and lower potentials, respectively. This may be attributed to the elevated scan rates resulting in enhanced electrical polarization effects on the electrodes [37,38]. The comparative CV curves at 200 mV/s of the CoS_2_ and Co_9_S_8_ symmetric SC cell are each plotted in Figure 7d. At 200 mV/s, Co_9_S_8_ displayed the largest CV curve compared to CoS_2_, indicating its excellent charge-storage capability as well as higher specific gravimetric capacitance.

**Figure 8:**
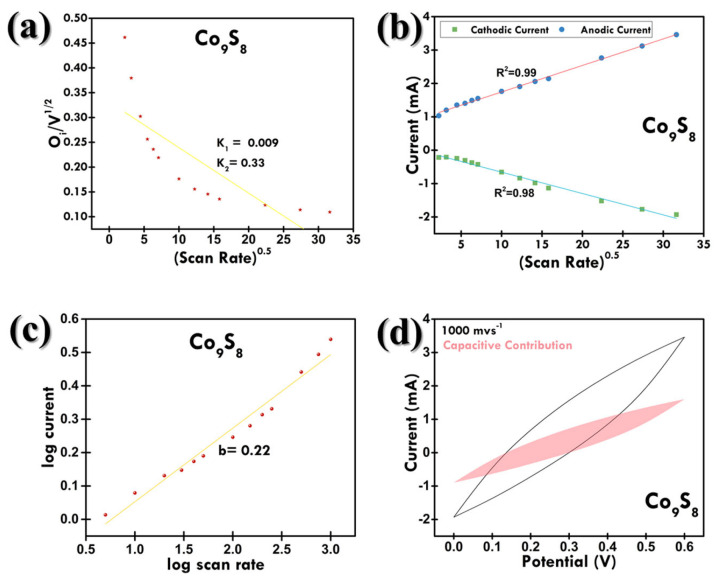
(**a**) Determination of k-value, (**b**) v^0.5^ vs. current plot for Co_9_S_8_, (**c**) b-value plot for Co_9_S_8_, and (**d**) capacitive contribution CV plot for Co_9_S_8_ at 1000 mV/s.

A linear relationship between current and (scan rate) v^0.5^ for Co_9_S_8_, as depicted in Figure 8b, suggests a diffusion-controlled process in accordance with the Randles–Sevcik equation [39] as follows:

(1) $$i_{p}=2.69\;\times \;{\mathrm{10}}^{5}n^{\mathrm{3/2}}{{\mathrm{AD}}_{0}}^{\mathrm{1/2}}v^{\mathrm{1/2}}C_{0}$$

where $i_{p}$ represents the peak current, n is the number of electrons transferred, A is the active surface area of the electrode, D_0_ is the diffusion coefficient of the rate-limiting protons, v is the scan rate, and C_0_ is the concentration of protons. On the other hand, for an adsorption-controlled process, the relation between peak current and scan rate should be linear, which is not what we see in Figure 8b. Thus, it is evident that the charge transfer kinetics is controlled by diffusion rather than adsorption. The diffusion coefficient observed for the anodic and cathodic current was around 0.07 and 0.06, indicating faster ion diffusion. The charge-storage mechanism in Co_9_S_8_ can be understood by plotting a log i vs. log v graph (Figure 8c), obeying the power law [40]:

(2) $$i=av^{b}$$

where i and v are current (mA) and scan rate (mV/s), respectively, and both a and b are constants. In our analysis, the electrochemical behavior was deconvoluted using the power-law relationship where the b-value indicates the dominant charge-storage mechanism. A b-value approaching 1 suggests a surface-controlled capacitive process, while a value closer to 0.5 is indicative of a diffusion-controlled process, associated with ion intercalation into the bulk of the electrode material [41]. We further applied Dunn’s method to quantitatively separate the capacitive (surface adsorption) and diffusion-limited (bulk intercalation) contributions to the total current. The observed high capacitive contribution, particularly in Co_9_S_8_, implies that a substantial portion of the charge storage arises from rapid surface redox reactions, which is beneficial for achieving high power density and excellent rate capability.

For Co_9_S_8_, the b value of 0.22 indicates the diffusion-controlled process. The diffusion-controlled process is ascribed to the profound faradaic reaction resulting from the diffusion of electrolyte ions into the bulk of the electrode [42]. The contribution rate of this process can be determined by the Dunn method, which follows the following equations:

(3) $$i=k_{1}v+k_{2}v^{0.5}$$

(4) $${i/v}^{0.5}=k_{1}v^{0.5}+k_{2}$$

**Figure 9:**
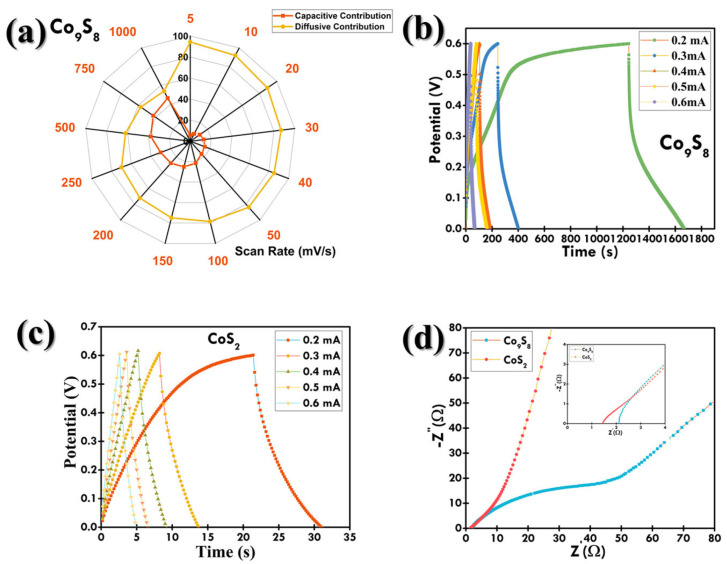
(**a**) Radar plot depicting capacitive and diffusive contribution at different scan rates for Co_9_S_8_; GCD plots of SC devices at various current values for (**b**) Co_9_S_8_ and (**c**) CoS_2_; (**d**) Nyquist plot for Co_9_S_8_ and CoS_2_ electrodes and zoomed view of Nyquist plot in inset.

The terms $k_{1}v$ and $k_{2}v^{0.5}$ indicate the current response generated at a specific potential as a result of diffusion-limiting and diffusion-controlled processes, respectively. The values of *k_1_* and *k_2_* can be determined by calculating the slope and y-intercept of the graph plotted, ${i/v}^{0.5}$ against $v^{0.5}$ for the device, as shown in Figure 8a. The CV curve of the diffusion-limited current contribution of the Co_9_S_8_ electrode at the scan rate of 1000 mV/s is shown in Figure 8d. It could be observed that the diffusion-controlled current contribution (53.69%) is higher than the diffusion-limiting current contribution (46.30%), indicating the pseudocapacitance mechanism even at high scan rate of 1000 mV/s. The diffusion-controlled contribution was maximum at 5 mV/s (94.25%). As the scan rate increased from 5 mV/s to 200 mV/s, the diffusion-controlled contribution decreased up to 72% (Figure 9a). As mentioned, this can be attributed to the sluggish ion movement compared to the scan rate, causing a high percentage of faradaic reactions to occur on the electrode surface, resulting in an increase in the diffusion-limiting current contribution.

**Figure 10:**
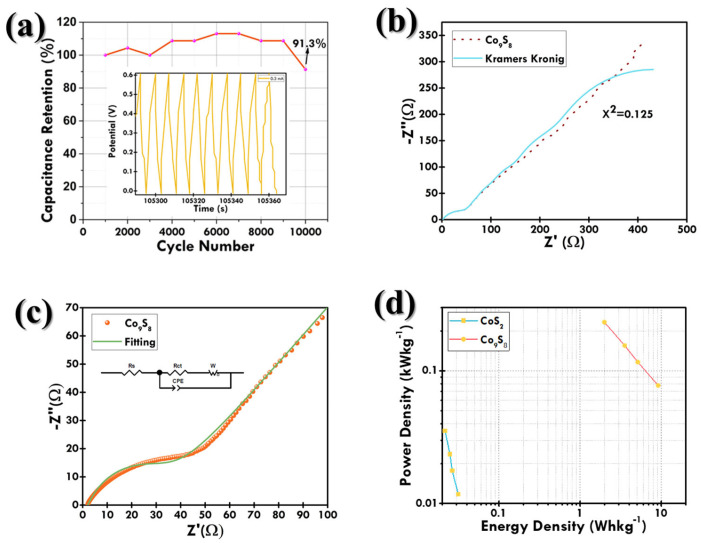
(**a**) Cycle stability test for Co_9_S_8_ SC device for 10,000 cycles at 0.3 mA; (**b**) KKT analysis for the validity of EIS experiment; (**c**) fitting of Co_9_S_8_ Nyquist plot using equivalent circuit; and (**d**) Ragone plot.

**Figure A2:**
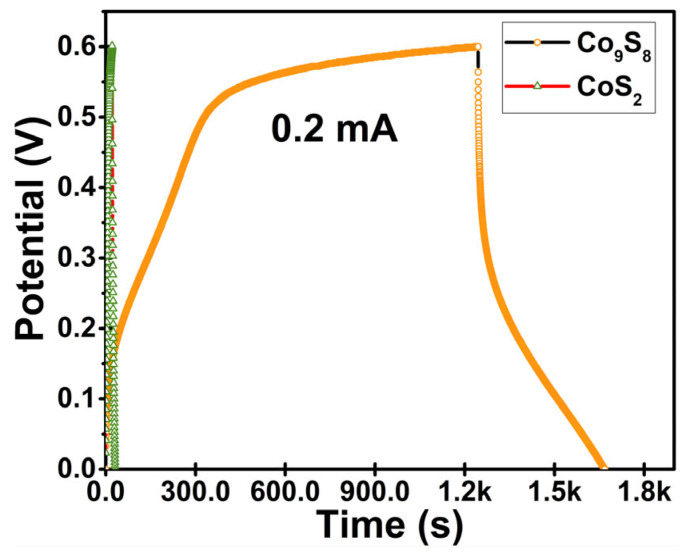
Comparative GCD plots of Co_9_S_8_ and CoS_2_ at 0.2 mA.

**Figure A3:**
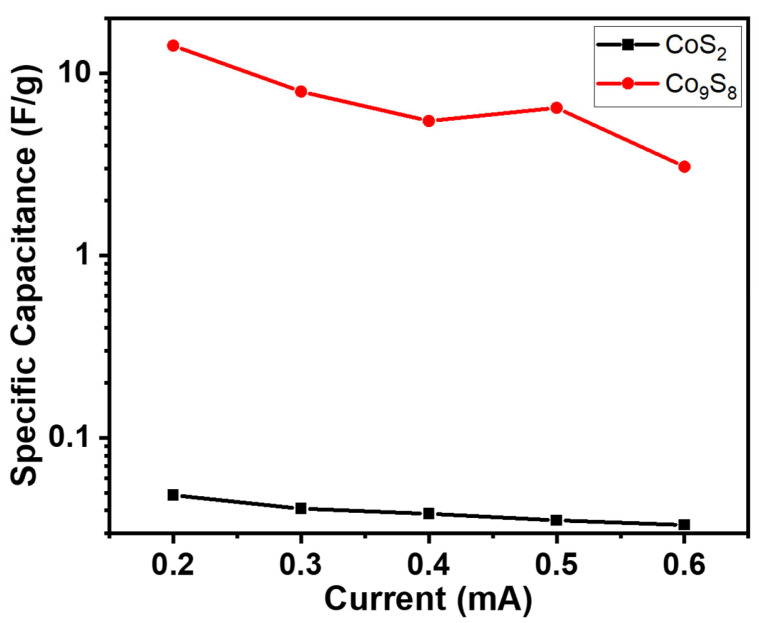
Specific capacitance vs. current plot of Co_9_S_8_ and CoS_2_.

The GCD plots at the 0.2 to 0.6 mA current values of CoS_2_ and Co_9_S_8_ are depicted in Figure 9b,c, respectively. These electrodes exhibited pseudocapacitor-type characteristics by showing non-linear charging–discharging curves due to the involvement of faradaic redox reactions [43], which is in accordance with the CV results. Furthermore, a decrease in discharging time is evident with the increase in current. The duration for OH^–^ ion intercalation into the electrode surface diminished with increasing current, leading to surface-confined faradaic reactions [44,45]. The comparative GCD curve at 0.2 mA (lowest) of both CoS_2_ and Co_9_S_8_ is plotted in Figure A2. The Co_9_S_8_ cell revealed the longest discharging time at both the lowest and highest currents, indicating its highest C_cell_ [46,47]. The C_cell_ of all devices from GCD was calculated using Equation (A1), and their capacitance at each current value is listed in Table A1. From Table A1, it can be depicted that Co_9_S_8_ showed superior C_cell_. The capacitance vs. current plot for both cells is depicted in Figure A3. In addition, the capacitance retention test was also performed for Co_9_S_8_, and the cell went through 10,000 continuous charging–discharging cycles at 0.3 mA. A decrease in capacitance was observed for the first 2000 cycles, which can be attributed to the saturation of the active site of the Co_9_S_8_ electrode during the beginning of the charging–discharging process [48]. Then, the capacitance was seen to increase to 6000 cycles, which can be attributed to the activation of the Co_9_S_8_ electrode by uninterrupted ion diffusion into the electrode material [49]. The initial fluctuations in specific capacitance during the early cycles are attributed to the electrode activation process, wherein the electrolyte progressively infiltrates into the porous structure of the electrode material, gradually exposing previously inaccessible electroactive sites [50]. This activation typically results in an initial increase or oscillation in capacitance values as the electrochemical interface stabilizes [51]. Subsequent minor fluctuations may also arise due to structural relaxation or reconstruction of the active material under repeated cycling. This includes changes in crystallinity, phase transitions, or partial dissolution/re-deposition phenomena that temporarily alter ion transport and redox activity. Additionally, electrode–electrolyte interfacial equilibrium can shift during prolonged cycling, contributing to transient performance variation before reaching a saturation regime. These effects are commonly reported in transition metal sulfides and oxides used in supercapacitors, especially during extended cycling at constant current densities. After that, the capacitance was seen to be decreased till the 10,000 cycle. It was observed that even after 10,000 GCD cycles the device maintained a capacitance of around 91.3%, as depicted in Figure 10a. This can be attributed to the degradation of the active material due to the continuous charging–discharging cycles [52].

To determine the validity of the EIS experiment, Kramers–Kronig transforms (KKT), a mathematical relationship for the real and imaginary components of a complex system, were implemented on the data [53,54]. The equations assure compliance with the principles of linearity, causality, and stability within the system. The real part of the impedance Z′(ω) is related to the imaginary part Z″(ω), with ω representing the angular frequency. KKT relations for EIS data are in accordance with the following equations:

(5) $$Z^{\prime }\left(\omega \right)=Z^{\prime }\;\left(\infty \right)+\frac{2}{\pi }\;\int_{0}^{\infty }\frac{\omega^{\prime }Z^{\prime \prime }(\omega \prime )}{{\omega \prime }^{2}-\omega^{2}}\;d\omega^{\prime }$$

(6) $$Z”\;\left(\omega \right)=-\frac{2\omega }{\pi }\;\int_{0}^{\infty }\frac{Z^{\prime }\left(\omega^{\prime }\right)-Z^{\prime }(\infty )}{{\omega \prime }^{2}-\omega^{2}}\;d\omega^{\prime }$$

where Z′(∞) = the real part of the impedance at infinite frequency. To determine the quality of fit, an *χ*^2^ value of 0.125 was obtained between the experimental and fitted data, as shown in Figure 10b, indicating KKT relations are well satisfied [55]. Figure 9d plots the EIS Nyquist curves of the Co_9_S_8_ and CoS_2_ electrodes. In the higher frequency regime, the x-intercept corresponds to series resistance (R_s_) and the semicircle diameter corresponds to charge-transfer resistance (R_ct_) [56]. The Nyquist plot was fitted according to the equivalent circuit, as shown in Figure 10c. The analysis shows a good fitting of the data.

The CoS_2_ electrode had the R_s_ and R_ct_ values of 1.349 and 4.174 × 10^3^ Ω, respectively, compared to Co_9_S_8_ having values of 1.986 and 2.824 × 10^1^ Ω, indicating the higher electrical conductivity of CoS_2_ [57,58]. Also, at the lower frequency regime of Nyquist plot, the steepness of the straight line signifies the diffusion behavior of electrolyte ions into the electrode surface, known as Warburg impedance (Z_w_) [59,60]. It can be seen that for CoS_2_ (1.132 × 10^−2^ Ω/Hz^1/2^), the Warburg impedance is steeper than for Co_9_S_8_ (8.436 × 10^−3^ Ω/Hz^1/2^), indicating the slower ion diffusion and higher impedance for CoS_2_. It indicates better ion diffusion for Co_9_S_8_, and the overall impedance is lower. This suggests that Co_9_S_8_ is also influenced by charge transfer rather than being purely diffusion-controlled.

The energy and power density of the device were calculated using Equations (A2) and (A3). Co_9_S_8_ shows the highest energy density of 9.14 Whkg^−1^ and power density of 0.23 kWkg^−1^. Table A2 lists the comparison of energy and power density values of both electrodes at different current values. This work has been compared with several works that involved CoS_2_ and Co_9_S_8_ electrode materials and has been listed in Table 1, and the Ragone plots are plotted in Figure 10d. The obtained data of the Ragone plot signify the device has both high E and P, indicating a high energy-storage ability. Hence, the Co_9_S_8_ SC device exhibited a superior energy-storage ability compared to the reported devices, as listed in Table 1.

**Figure 11:**
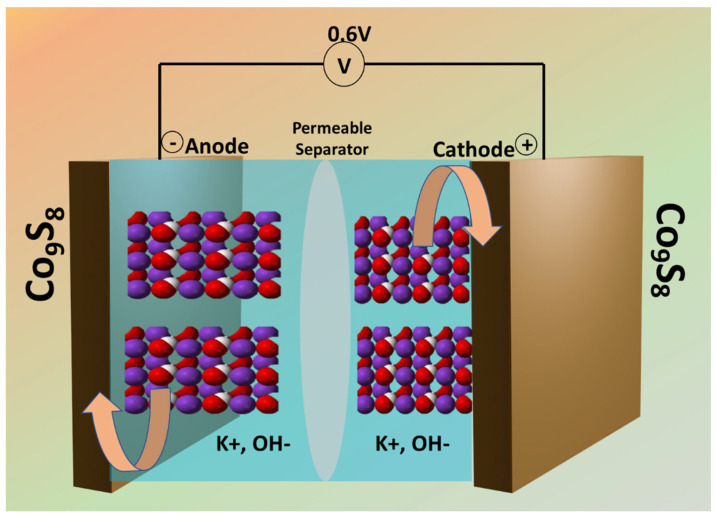
Schematic representation of charge-storage mechanism in Co_9_S_8_ symmetric SC device.

In the midst of the CoS_2_ and Co_9_S_8_ electrodes, Co_9_S_8_ showed the highest capacitance and capacity retention from CV and GCD measurements. The outstanding performance of the Co_9_S_8_ electrode can be related to the presence of multiple valence states and the specific coordination environment in Co_9_S_8_, providing a greater number of electrochemically active sites for faradaic reactions. The specific arrangement of Co and S atoms in Co_9_S_8_ may create stronger Co-S bonds, enhancing the materials structure stability during electrochemical cycling. This can result in higher electrochemical activity due to lower resistance to electron transfer. The arrangement of Co and S atoms due to the complex and unique crystal structure in Co_9_S_8_ creates a framework that enhances electron mobility and ion diffusion, leading to faster C/D cycles. Co_9_S_8_ exhibits cobalt in various oxidation states (Co^2+^, Co^3+^). The presence of these mixed valence states facilitates various pathways for redox reactions throughout the charge and discharge cycles, enhancing pseudocapacitive behavior. This range of redox states facilitates enhanced electron and ion exchange efficiency as shown in Figure 11. In contrast, CoS_2_ exhibits a more obvious valence-state distribution, predominantly featuring Co^2+^. This characteristic restricts the potential for redox reactions, consequently diminishing the overall capacitance and energy-storage capacity. Along with this, Co_9_S_8_ exhibits greater chemical stability than CoS_2_, particularly during repeated C/D cycles, leading to enhanced cycling stability and an extended operational lifespan for the SC. Although Co_9_S_8_ demonstrates commendable electrical conductivity, it generally does not surpass that of CoS_2_. Nonetheless, Co_9_S_8_ offsets this with enhanced electrochemical characteristics, such as increased redox activity-improved structural stability and a greater number of active sites for faradaic reactions. This explains why Co_9_S_8_ excels compared to CoS_2_ regarding overall electrochemical performance, even though it has comparatively lower electrical conductivity.

## 4. Conclusions

Hierarchical and multiphase structures of Co_x_S_y_ with different morphologies were successfully synthesized via a simple one-pot hydrothermal method. The morphology of the samples was controlled by a binary solvent mixture. Among the two, the Co_9_S_8_ electrode exhibited excellent electrochemical performance. The Co_9_S_8_ electrode exhibited the highest capacitance calculated from a GCD measurement. The Co_9_S_8_ symmetric SC device showed the highest capacitance of 14.12 Fg^−1^, with the highest E and P density values of 9.14 Whkg^−1^ and 0.23 kWkg^−1^. Moreover, the device showed an excellent capacitance retention of 91.3% after 10,000 cycles. These can be explained by the effect of greater surface activity, mixed valence states, stronger bonding, and superior electron and ion mobility, creating a synergistic effect in Co_9_S_8_. Hence, the Co_9_S_8_-based pseudocapacitor shows excellent electrochemical properties for SC applications. Further improvement can be made by making heterostructures with other excellent layered structure conductive materials (e.g., MXene, graphene, TMDs), increasing the surface area for the growth of electrode material. This promotes an even larger surface area for faradaic reactions and further improves the electrochemical performance.

## Figures and Tables

**Table 1:** Comparable performance of developed SC device with literature.

Electrode Material	Method	Capacitance (Fg^−1^)	Electrolyte	Substrate	Cycle Life	Ref
Co_9_S_8_/CHS	In situ carbonization	113.02	6 M KOH	Ni-F	90% (2000)	[61]
CoNi_2_S_4_/Co_9_S_8_	Hydrothermal	1546.5	6 M KOH	-	87% (10,000)	[62]
Co_9_S_8_@RGO	Hydrothermal	3255	PVA/KOH	Ni-F	80.61 (10,000)	[63]
NiCo_2_S_4_/Co_9_S_8_	Hydrothermal	2532.5	3 M KOH	Ni-F	94.7% (10,000)	[64]
Mn-Co_9_S_8_	Self-templating sulfurization	234.6	PVA/KOH	Ni-F	98.2% (5000)	[65]
NiCo_2_S_4_/[Ni, Co]_9_S_8_	Hydrothermal	1789	6 M KOH	Ni-F	70% (3000)	[66]
Co_9_S_8_ nanotube	Hydrothermal	285.3	6 M KOH	Ni-F	90.4% (1000)	[67]
MnCo_2_S_4_/Co_9_S_8_	Hydrothermal	1100.5	6 M KOH	Ni-F	94.8% (5000)	[68]
rGO/Ni_3_S_2_/Co_9_S_8_	Solution-based method	1929.1	2 M KOH	Ni-F	92.8% (1000)	[69]
Co_9_S_8_	Hydrothermal	14.12 at 0.2 mA	4M KOH	Ni-F	91.3% (10,000)	Present Work

## Data Availability

The raw data supporting the conclusions of this article will be made available by the authors on request.

## Appendix A

Equations

(A1) $$C_{\mathrm{cell}}\;(F.g^{-1})=\frac{i\times ∆t}{m\times ∆V}$$

(A2) $$E_{d}\;(\mathrm{Wh}.kg^{-1})=\frac{C_{cell}(F.g^{-1})\times ∆V^{2}}{2\times 3.6\;}$$

(A3) $$P_{d}\;(\mathrm{kW}.kg^{-1})=\frac{E_{d}(Wh.kg^{-1})\times 3.6}{\;∆t}$$

Here, m(g) is the mass loading of any one electrode, M(g) = 2m is the total mass of the whole device, i.e., the mass of both the electrodes in the device combined, ∆V(V) is the device voltage window, and ∆t(s) is the discharging time.

**For CoS_2_:**

**During charging (oxidation):**

CoS_2_ + OH^−^ → CoS_2_(OH) + e^−^ (A4)

**During discharging (reduction):**

CoS_2_(OH) + e^−^ → CoS_2_ + OH^−^ (A5)

**For Co_9_S_8_:**

**During charging (oxidation):**

Co_9_S_8_ + OH^−^ → Co_9_S_8_(OH) + e^−^ (A6)

**During discharging (reduction):**

Co_9_S_8_(OH) + e^−^ → Co_9_S_8_ + OH^−^ (A7)

**Figures**

**Table A1:** C_cell_ of all devices from GCD.

Current (mA)	CoS_2_	Co_9_S_8_
	C_cell_ (F/g)	C_cell_ (F/g)
0.2	0.048	14.12
0.3	0.040	7.91
0.4	0.038	5.45
0.5	0.035	6.43
0.6	0.033	3.06

**Table A2:** Specific energy density and specific power density calculated for CoS_2_ and Co_9_S_8_ from GCD.

Current (mA)	CoS_2_		Co_9_S_8_	
	Specific Energy Density (Wh/kg)	Specific Power Density (W/kg)	Specific Energy Density (Wh/kg)	Specific Power Density (kW/kg)
0.2	0.031	0.011	9.14976	0.07776
0.3	0.02	0.017	5.12568	0.11664
0.4	0.024	0.023	3.53376	0.15552
0.5	0.022	0.029	4.1688	0.1944
0.6	0.021	0.035	1.98288	0.23328
